# Supplementary material for: The YBR056W-A and Its Ortholog YDR034W-B of S. cerevisiae Belonging to CYSTM Family Participate in Manganese Stress Overcoming
Source: Genes (Basel). 2023 Apr 27;14(5):987. doi: 10.3390/genes14050987 (PMC10217909; doi:10.3390/genes14050987)
Supplement: Supplementary file 1 [file genes-14-00987-s001.zip › genes-2332052-supplementary.pdf]

Supplementary Figure S1

The cell count and mean X (characterizing the fluorescence intensity) for the cells of *YDRO34W-B-GFP* and *YBR056W-A-GFP* strains of *S. cerevisiae* cultivated for 24 h in control YPD (C), and in YPD supplemented with 0.2 mM Cd^{2+} (Cd) or 4 mM Mn^{2+} (Mn)

The cell concentration in the cultivation medium was determined by flow cytometry. After a series of dilutions of the cell suspension with water, the number of cells in 25 µl in each sample was counted on a NovoCyte Flow cytometer (Agilent, USA). Expression of GFP-tagged proteins was determined on a NovoCyte flow cytometer using 488 nm for excitation and 585 nm for emission. 100 000 cells were counted at each experimental point. All assays were repeated 4–5 times and the representative cells count curves are presented.

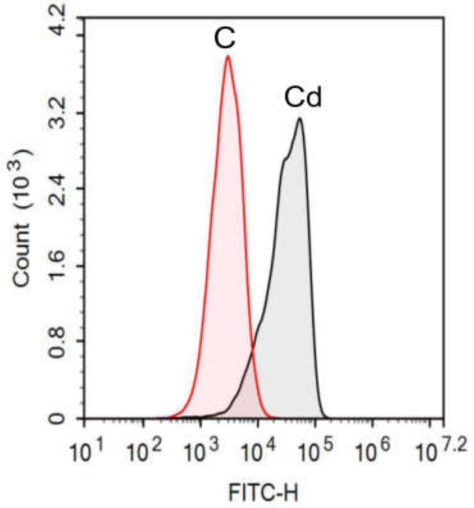

YDR034W-B-GFP

Mean X (Cd): 40,348

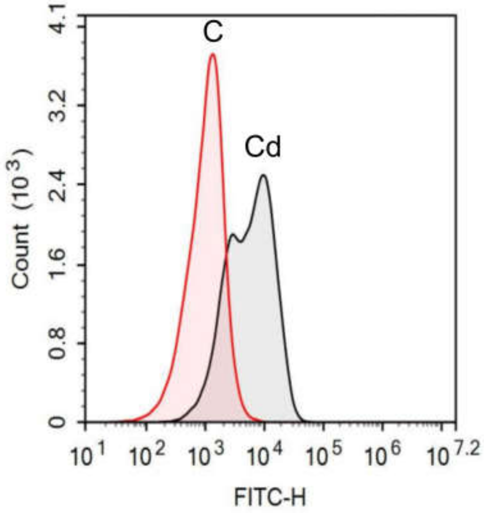

YBR056W-A-GFP

Mean X (Cd): 8,542

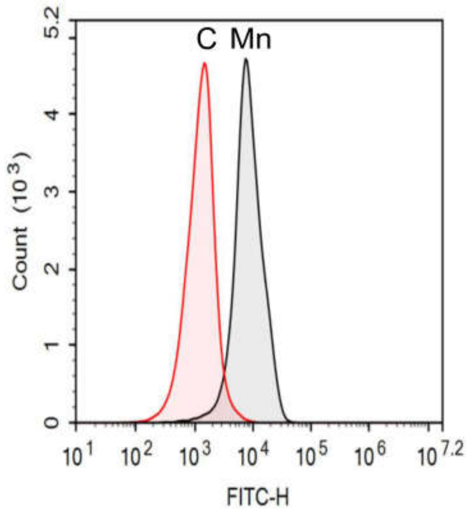

YDR034W-B-GFP

Mean X (Mn): 10,013

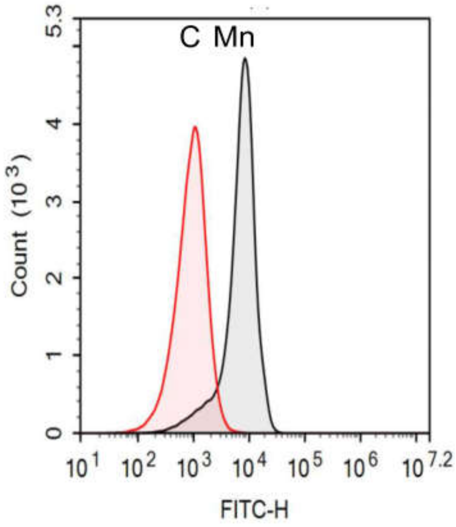

YBR056W-A-GFP

Mean X (Cd): 8,47
